# Tumor-Derived Exosome FGD5-AS1 Promotes Angiogenesis, Vascular Permeability, and Metastasis in Thyroid Cancer by Targeting the miR-6838-5p/VAV2 Axis

**DOI:** 10.1155/2022/4702855

**Published:** 2022-04-29

**Authors:** Bo Liu, Jiaming Chen, Fangjian Shang, Meng Lian, Xixi Shen, Jugao Fang

**Affiliations:** ^1^Department of Otorhinolaryngology Head and Neck Surgery, Beijing Tongren Hospital, Capital Medical University, Beijing 100730, China; ^2^Department of General Surgery, The First Hospital of Hebei Medical University, Shijiazhuang, Hebei 050031, China

## Abstract

Exosomes are small vesicles with a diameter of 30~150 nm secreted by cells, which are rich in mRNA, microRNA, and long noncoding RNA (lncRNA). The biological functions of most exosomal lncRNAs are not well understood. Studies have shown that tumor exosome FGD5-AS1 plays an important role in the proliferation, migration, and invasion of tumor cells. In this study, SW1736 and KAT18 TC cells with high expression of FGD5-AS1 were screened. Exosomes with high expression of FGD5-AS1 were collected. The collected exosomes were then added to HUVEC cells. After incubation for 24 h, the effects on the proliferation and migration of HUVEC cells and vascular permeability were detected. The results showed that TC cells SW1736 and KAT18 could secrete a large number of exosomes, which could be taken up by HUVEC cells. Overexpression of FGD5-AS1 enhanced proliferation, migration, angiogenesis, and permeability of HUVEC. This effect is achieved through activation of the miR-6838-5p/VAV2 axis. These results suggest that FGD5-AS1 in tumor-derived exoskeleton promotes angiogenesis, vascular permeability, and metastasis by regulating the endothelial miR-6838-5p/VAV2 axis and ultimately promotes the occurrence and development of TC.

## 1. Introduction

Thyroid cancer (TC) is the most common endocrine malignant tumor [1]. In recent years, the incidence of the disease has gradually increased [2]. The pathogenesis of TC is complicated, and no consensus has been reached at present [3]. The pathogenesis is related to radioactive radiation, abnormal iodine intake, and genetic changes of BRAF, p53, hMLH1, and hMSH2 genes [4]. Tumor cells induce angiogenesis. Tumor cells establish a special and complex matrix microenvironment of a cytokine network to promote tumor growth [5]. In this way, tumor-related blood vessels and lymphatic vessels can be induced continuously, providing the factors for the interaction between tumor cells and stromal cells [6].

Exosomes are small membranous vesicles (30-150 nm) secreted by most cells and play an important role in intercellular communication [7]. Many noncoding RNAs (ncRNAs), such as long noncoding RNAs (lncRNAs), initiate tumorigenesis and EMT processes and promote tumor angiogenesis [8]. Exosomes can carry lncRNAs to local and distal cells. lncRNAs play a key role in gene regulation at epigenetic, transcriptional, and posttranscriptional levels. lncRNA directly or indirectly affects the occurrence and progression of cancer, and its function is related to subcellular localization and targets [9]. lncRNA is highly enriched in exosomes. lncRNAs coated by exosomes are not degraded by RNA enzymes and can exist stably in various body fluids. lncRNAs of tumor cells act on cell components in tumor microenvironment through exosomes and promote tumor progression from multiple perspectives. FGD5-AS1 has a wide range of biological effects and is associated with the development of a variety of tumors. Studies have shown that FGD5-AS1 upregulates CDCA7 and promotes malignant evolution of colorectal cancer cells [10]. In addition, it also plays a role in oral cancer [11], glioma [12], gastric cancer [13], non-small-cell lung cancer [14], melanoma [15], and other cancers. However, the role of FGD5-AS1 in TC, especially in metastatic TC, has not been reported.

VAV2, a member of the VAV family of proteins [16, 17], is expressed in almost all tissues and detected at various developmental stages [17]. VAV2 is involved in cell morphology and actin cytoskeleton organization and formation in cells [18]. It also participates in signal transduction of growth factors to the cytoskeleton. It also plays an important role in angiogenesis and nitric oxide-mediated vasodilation [19]. In recent years, members of the Vav gene family have been found to be closely related to malignant tumors. However, so far, the study on the relationship between Vav2 and TC is not comprehensive.

This study intends to investigate the role of exosome-mediated FGD5-AS1 in the development and progression of TC and provide a new direction for clinical diagnosis and treatment of TC.

## 2. Methods

### 2.1. Cell Lines and Human Tissue Samples

TC lines SW1736 and KAT18 and normal thyroid cells NTHY-ORI3-1 were cultured in RPMI-1640 containing 10% FBS. The cell lines were purchased from American Type Culture Collection (ATCC, Manassas, VA, USA). 10% fetal bovine serum (FBS), 100 U/mL penicillin, and 100 *μ*g/mL streptomycin were added. Cells were cultured in an incubator containing 5% CO_2_ at 37°C. When cells reached 70% density, medium RPMI-1640 without FBS was replaced. After starvation for 48 h, the cell supernatant was collected and stored at -80°C until use. Open thyroid surgery was used to collect TC and paracancer tissues with pathologically confirmed size of 0.5 cm × 0.5 cm. Morning fasting forearm venous blood of TC patients was extracted 1 d before surgery, and exosomes were separated for use. The study was approved by our ethics Committee of Beijing Tongren Hospital.

### 2.2. Cell Transfection

Proper amount of cells was inoculated in 25 mm^2^ petri dish. After 80% fusion, the synthesized overexpressed plasmid and control were transferred into cells mediated by Lipofectamine 2000 and followed the instructions of the liposome reagent. The experiment was repeated three times.

### 2.3. RT-qPCR

Total RNA was extracted from cells according to the instructions of the Trizol kit (Invitrogen, USA). 1 *μ*g of total RNA was reverse transcribed into cDNA using the RNA reverse transcription kit (Prime Script™ RT reagent Kit, TaKaRa, Japan). The reaction volume was 20 *μ*L. PCR amplification was performed according to the PCR kit with *β*-actin as the internal reference. Reaction conditions were as follows: 95°C for 5 min, 95°C for 20 s, 60°C for 30 s, and 72°C for 30 s, a total of 40 cycles. The upstream sequence of miR-6838-5p is GCACTCCTGGATGCCAATCT, and the sequence downstream is CTCTACAGCTATATTGCCAGCCAC. The relative expression was calculated using the 2 − ^△△Ct^ method.

### 2.4. Western Blot

Total cell protein was extracted, and protein concentration was detected. After sodium dodecyl sulfate- (SDS-) polyacrylamide gel electrophoresis (PAGE), the protein samples were transferred to the polyvinylidene fluoride (PVDF) membrane. The blocking solution was sealed at room temperature for 1 h, and a primary antibody (1 : 1000) was added and incubated overnight at 4°C. A second antibody (1: 2000) was added and incubated for 2 h, and the gray value of protein bands was detected with Quantity One software. The ratio of the target band to the reference band was used as the protein expression level.

### 2.5. Extraction and Identification of Exosomes

The extraction procedure of exosomes was as follows: cell supernatant at 2000 × *g* and centrifugation at 4°C for 30 min. The supernatant was collected at 10000 × *g* and centrifuged at 4°C for 30 min. Finally, the supernatant was centrifuged at 110000 × *g* at 4°C for 70 min. The precipitate was suspended in 2 mL 1× PBS. Filter with a 0.22 *μ*m aperture filter. Centrifugation was continued at 110000 × *g* at 4°C for 70 min. Discard the supernatant, 50~100 *μ*L, 1× PBS, and resuspend for later use. Exosome identification was performed using transmission electron microscopy (TEM): 20 *μ*L exosomes were added to the copper wire for adsorption for 1 min, and the excess liquid was sucked out with filter paper. Then, the exosomes adsorbed on the copper net were negatively stained with 2% (*w*/*v*) phosphotungstic acid (pH 6.8) for 2 min. Use filter paper to soak up excess fluid. The exosomes were dried under incandescent lamp and analyzed by TEM (Transmission Electron Microscope) (Philips, Japan).

### 2.6. In Vivo Swallowing Detection of Exosomes

Endocytic exosomes were stained with PKH26 (Sigma-Aldrich, USA) and followed instructions. The stained exosomes were added into HUVEC cell culture medium and incubated for 24 h and then detected by laser confocal microscopy.

### 2.7. Double-Luciferase Reporter Gene

FGD5-AS1 wild-type and mutated luciferase expression vectors (WT-FGD5-AS1 and MUT-FGD5-AS1) containing miR-6838-5p binding sites were constructed and cotransfected into KAT18 cells with miR-con and miR-6838-5p, respectively. Luciferase activity was detected according to the instructions, and the experiment was repeated 3 times.

### 2.8. CCK-8

Cells at the logarithmic growth stage were inoculated into 96-well plates. There were 1000 cells in each well and 5 multiple pores in each well. Culture them in a 37°C 5%CO_2_ incubator. Add 10 *μ*L CCK⁃8 solution to each well until the cells adhere to the wall. After incubation in the incubator for 2.5 h, the OD450 value of each well was detected on the multifunctional microplate tester. Cell activity was plotted by using the obtained OD450 as the ordinate and the detection time as the abscissa.

### 2.9. Transwell Experiment

2 × 10^4^ cells, 200 *μ*L in total, were resuspended in FBS-free medium 1640. Place them on the upper layer of each chamber (BD, USA). Cells were placed in 600 *μ*L medium 1640 containing 10% FBS. Culture them at 5% CO_2_ and 37°C. After 24 h, the cells were collected and fixed with 0.1% crystal violet for 10 min. After washing, the cells in the upper compartment were dried with cotton swabs, and 5 fields were taken under an inverted microscope (Olympus, Japan) and counted.

### 2.10. Immunofluorescence Staining

Cells were cultured in a 6-well plate covered with cell slivers. When the cells grew and fused to 90%, the culture medium was discarded. The cells were fixed with 4% paraformaldehyde on ice for 20 min and then covered with 0.1% Triton X-100 and stood at room temperature for 5 min. The primary antibody diluted with 1% bovine serum albumin (VE-cadherin 1 : 200 diluted) was added and incubated overnight at 4°C and then washed. A PBS-diluted fluorescent secondary antibody (1 : 400) was added and incubated at room temperature away from light for 60 min. DAPI covered the cell surface and stood at room temperature in the dark for 2 min. Seal the sheet and store at 4°C away from light for later use.

### 2.11. Experiments on Animals

The culture medium of cancer cells in the logarithmic growth phase was discarded, and the cells were washed with sterile neutral PBS once. Trypsin digestion and elution from a culture flask blow into single-cell suspension. After centrifugation, the density was about 5 × 10^7^/mL. 0.2 mL single-cell suspension was inoculated subcutaneously on the right back of nude mice. After inoculation, the growth of the tumor at the inoculation site was observed closely. In this study, the long and short diameters of tumors in nude mice were measured using Vernier calipers. The tumor volume calculation formula is volume = long axis × short axis^2^/2. On day 28, the nude mice were euthanized and tumor tissues were collected. The nude mice were euthanized by carbon dioxide in accordance with animal ethical welfare requirements. Carefully remove the fiber envelope and blood vessels on the surface, rinse with PBS buffer solution, and put at -80°C for later use.

### 2.12. Statistical Analysis

All experiments were repeated three times. SPSS 18.0 software was used for statistical analysis. The *t*-test was used to compare the differences between the two sample means. Comparison between multiple groups was performed by one-way ANOVA. The Pearson correlation coefficient was employed to calculate coexpression correlations. The Kaplan-Meier method was used for survival analysis. ^∗^*P* < 0.05 was considered significant and statistically significant.

## 3. Results

### 3.1. Exosome FGD5-AS1 Is Abundant in TC Patients

**Figure 1:**
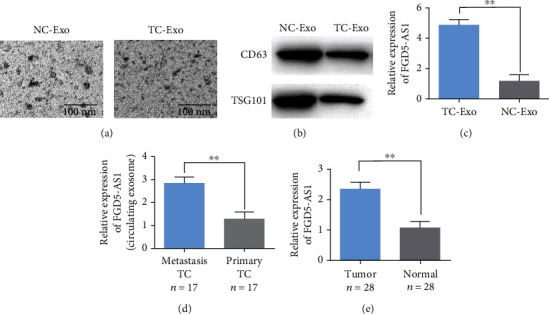
Exosomal FGD5-AS1 is enriched in TC patients. (a) The morphology of exosomes was observed with a transmission electron microscope. (b) Western blot detection of exosomal markers CD63 and TSG101. (c) Expression levels of FGD5-AS1 in exosomes of TC cells and normal thyroid cell lines. (d) The expression level of FGD5-AS1 in plasma exosomes of TC patients. (e) The expression level of FGD5-AS1 in tumor tissue and adjacent tissue of TC patients. Data are presented as mean ± SD (^∗∗^*P* < 0.01).

The supernatant of culture medium of TC cells was collected, and exosomes were collected by centrifugation successively. When observed under TEM, exosomes presented spherical or elliptical vesicles (Figure 1(a)). Western blot detection of exosome markers CD63 and TSG101 is presented in Figure 1(b). The exosomes extracted from the above results have high purity and can be used for subsequent experiments. The expression level of FGD5-AS1 in exosomes of TC cells and normal thyroid cell lines showed that FGD5-AS1 was highly expressed in exosomes extracted from TC cells (Figure 1(c)). Results of real-time PCR showed that the expression of FGD5-AS1 in the serum exosome of patients with metastatic TC was higher than that of patients with lung metastatic TC (Figure 1(d)). In addition, the expression level of FGD5-AS1 in tumor tissues of TC patients was higher than that in adjacent tissues (Figure 1(e)).

### 3.2. FGD5-AS1 Secreted by TC Is Transferred to HUVECs

**Figure 2:**
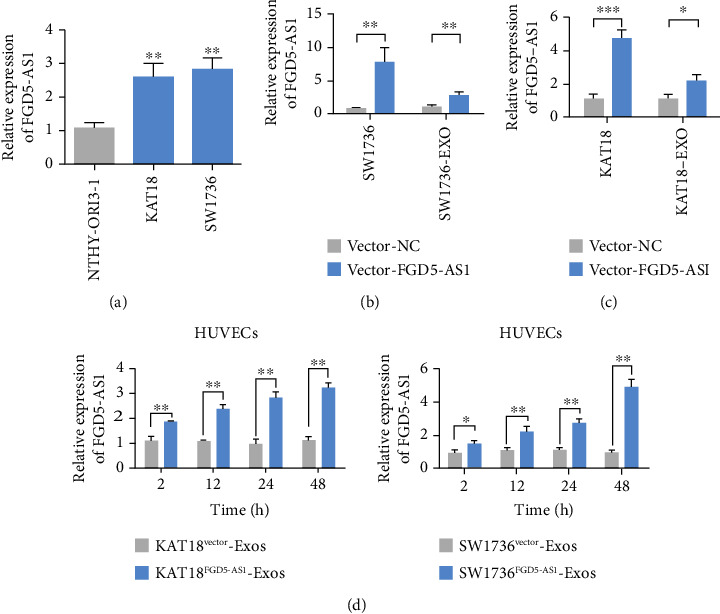
TC-secreted FGD5-AS1 metastasizes to HUVECs. (a) The level of FGD5-AS1 in the TC cell line was analyzed using qRT-PCR. (b) Expression of FGD5-AS1 in SW1736 cells transfected with vector-FGD5-AS1 or vector-NC and qRT-PCR detection of exosomes derived therefrom. (c) Expression of FGD5-AS1 in KAT18 cells transfected with vector-FGD5-AS1 or vector-NC and qRT-PCR detection of its derived exosomes. (d) qRT-PCR analysis of FGD5-AS1 expression in HUVECs incubated with exosomes from TC cells for 2, 12, 24, and 48 hours. Data are presented as mean ± SD (^∗^*P* < 0.05, ^∗∗^*P* < 0.01, ^∗∗^*P* < 0.001).

In order to screen TC cell lines with different FGD5-AS1 expression levels, this study used qRT-PCR to detect the expression levels of FGD5-AS1 in NTHY-ORI3-1, SW1736, and KAT18. The results showed that SW1736 and KAT18 cells had high expression of FGD5-AS1 (Figure 2(a)). The overexpressed FGD5-AS1 plasmid was transfected into SW1736 and KAT18 cells. The expression levels of FGD5-AS1 in SW1736 and KAT18 cells and their exosomes were detected by qRT-PCR. The results showed that the expression level of FGD5-AS1 in SW1736 and KAT18 cells and their exosomes transfected with the FGD5-AS1 overexpressed plasmid was higher than that in control cells and their exosomes (Figures 2(b) and 2(c)). After incubation with exosomes from TC cells for 2, 12, 24, and 48 h, FGD5-AS1 expression in HUVECs was detected by qRT-PCR. The results showed that with the extension of incubation time, the expression of FGD5-AS1 in HUVEC cells was upregulated (Figure 2(d)).

### 3.3. Overexpression of FGD5-AS1 Enhanced Proliferation, Migration, Angiogenesis, and Permeability of HUVEC

**Figure 3:**
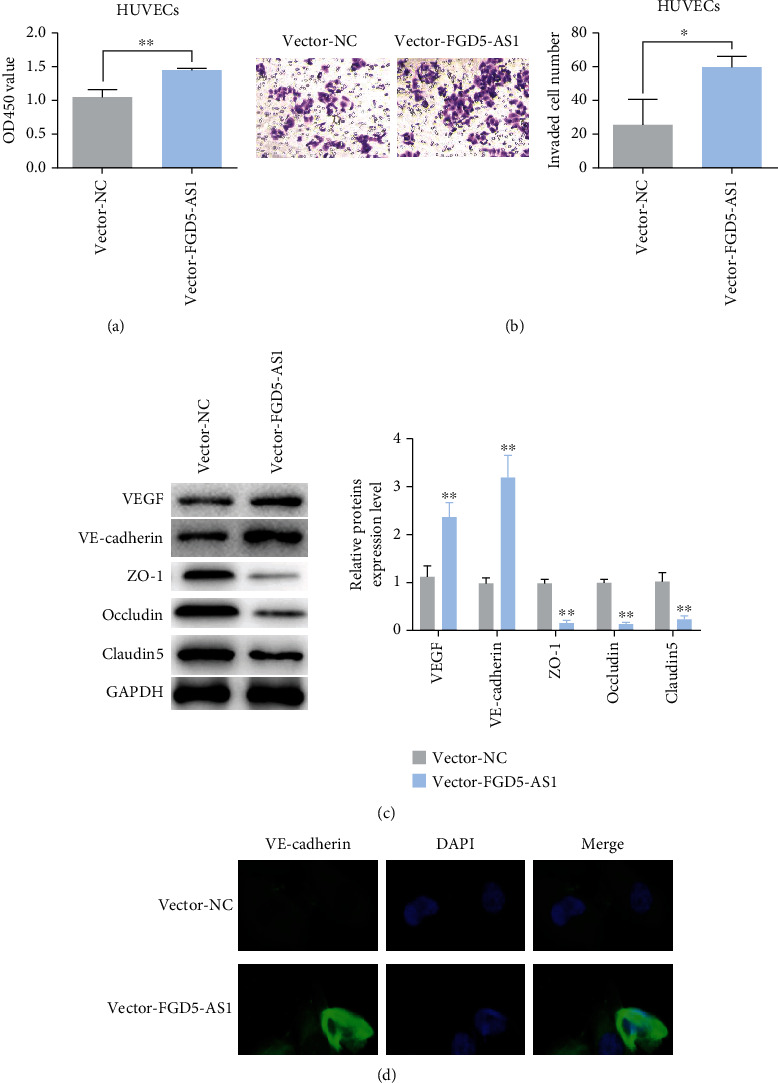
Overexpression of FGD5-AS1 enhances HUVEC proliferation, migration, angiogenesis, and permeability. (a) The proliferation of HUVECs after transfection with the FGD5-AS1 overexpression plasmid was detected by the CCK-8 method. (b) The effect of FGD5-AS1 on HUVEC migration was detected by the transwell method. (c) Western blot detection of VEGF, VE-cadherin, ZO-1, occludin, and claudin5 protein levels in HUVECs after overexpression of FGD5-AS1. (d) Immunofluorescence analysis of VE-cadherin expression after FGD5-AS1 transfection of HUVECs. Data are presented as mean ± SD (^∗^*P* < 0.05, ^∗∗^*P* < 0.01).

The proliferation of HUVECs after transfection with the FGD5-AS1 overexpressed plasmid was detected by the CCK-8 method. The results showed that the proliferation capacity of HUVEC cells was higher than that of the control group after the addition of the FGD5-AS1 overexpressed plasmid (Figure 3(a)). Cell migration experiment can reflect cell migration ability well. Transwell test results showed that after the addition of the FGD5-AS1 overexpressed plasmid, the ability of HUVEC cells to cross the chamber was higher than that of the control group (Figure 3(b)). Western blot detected the protein levels of VEGF, VE-cadherin, ZO-1, occludin, and claudin5 in HUVEC after overexpression of FGD5-AS1. The results showed that the expression levels of vascular markers VEGF and VE-cadherin were upregulated after overexpression of FGD5-AS1, while the expression levels of vascular permeability markers ZO-1, occludin, and claudin5 were downregulated (Figure 3(c)). Immunofluorescence staining results showed that the expression of VE-cadherin was enhanced after HUVEC transfection of FGD5-AS1 (Figure 3(d)). These results suggested that overexpression of FGD5-AS1 enhanced proliferation, migration, angiogenesis, and permeability of HUVEC.

### 3.4. miR-6838-5p Is the Target miRNA of FGD5-AS1

**Figure 4:**
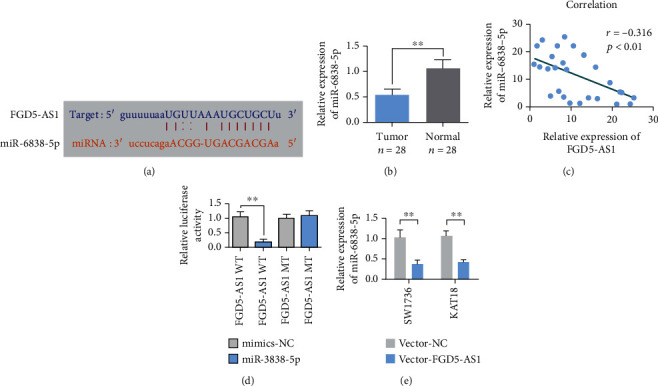
miR-6838-5p is an FGD5-AS1-bound miRNA. (a) FGD5-AS1 predicted the binding site to miR-6838-5p. (b) qRT-PCR detection of miR-6838-5p expression in 28 tumors and adjacent normal tissues. (c) Pearson correlation analysis showed a correlation between FGD5-AS1 and miR-6838-5p expression (*n* = 28). (d) FGD5-AS1 wild-type (wt) and mutant (mut) luciferase reporter vector experiments. (e) qRT-PCR detection of miR-6838-5p expression in SW1736 and KAT18 cells transfected with FGD5-AS1. Data are presented as mean ± SD (^∗∗^*P* < 0.01).

Bioinformatics techniques predicted the target gene of FGD5-AS1. StarBase software predicted that the target gene of FGD5-AS1 might be miR-6838-5p. The complementary sequence of miR-6838-5p and FGD5-AS1 mRNA is shown in Figure 4(a). PCR detection results showed that miR-6838-5p was highly expressed in TC tissues (Figure 4(b)). Pearson correlation analysis showed that there was a coexpression negative correlation between FGD5-AS1 and miR-6838-5p expression (Figure 4(c)). FGD5-AS1 was regulated by miR-6838-5p and verified by the double-luciferase reporter gene experiment, and the relative luciferase activity of wild-type plasmid+miR-6838-5p mimics was decreased compared with that of wild-type plasmid+NC mimics. Compared with the cotransfected mutant plasmid+NC mimics, there was no significant difference in the relative luciferase activity of the cotransfected mutant plasmid+miR-6838-5p mimics (Figure 4(d)), indicating that miR-6838-5p and FGD5-AS1 could target binding. qRT-PCR showed that overexpression of FGD5-AS1 could inhibit the expression of miR-6838-5p in SW1736 and KAT18 cells (Figure 4(e)).

### 3.5. Identification of VAV2 as the miRNA Target Oncogene

**Figure 5:**
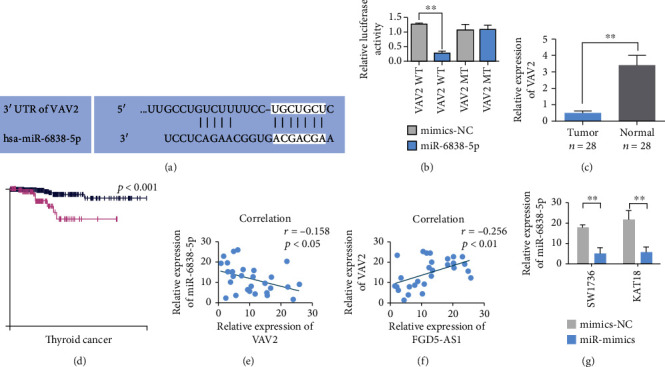
Identification of VAV2 as a miRNA target oncogene. (a) Predicted binding site information of miR-6838-5p to VAV2. (b) VAV2 wild-type (wt) and mutant (mut) luciferase reporter vector assays. (c) VAV2 is upregulated in TC tumors. (d) VAV2 expression is associated with poorer OS. The survival analysis data of VAV2 was downloaded from the open source database The Human Protein Atlas (http://www.proteinatlas.org/). (e) Pearson correlation analysis showed a correlation between miR-6838-5p and VAV2 expression (*n* = 28). (f) Pearson correlation analysis showing the correlation of FGD5-AS1 and VAV2 expression (*n* = 28). (g) qRT-PCR analysis shows that miR-6838-5p downregulates VAV2 in SW1736 and KAT18 cells. Data are presented as mean ± SD (^∗∗^*P* < 0.01).

The prediction of TargetScan software showed that the target gene of miR-6838-5p might be VAV2 (Figure 5(a)). Two luciferase reporter gene experiments verified the targeted regulation of miR-6838-5p on VAV2 mRNA showing that the relative luciferase activity of wild-type plasmid+miR-6838-5p mimics was decreased compared with that of wild-type plasmid+NC mimics. Compared with the cotransfected mutant plasmid+NC mimics, the relative luciferase activity of cotransfected mutant plasmid+miR-6838-5p mimics showed no statistical significance (Figure 5(b)). The expression level of VAV2 in tumor tissues of TC patients is higher than that in adjacent tissues (Figure 5(c)). In addition, patients with high VAV2 expression had poor prognosis and short survival time (Figure 5(d)). Pearson correlation analysis showed that there was a coexpression negative correlation between VAV2 and miR-6838-5p expression (Figure 5(e)). There was a positive coexpression correlation between VAV2 and FGD5-AS1 (Figure 5(f)). qRT-PCR showed that after overexpression of miR-6838-5p, VAV2 expression was decreased in SW1736 and KAT18 cells (Figure 5(g)).

### 3.6. Exosome FGD5-AS1 from TC SW1736 and KAT18 Cells Promotes Angiogenesis In Vitro

**Figure 6:**
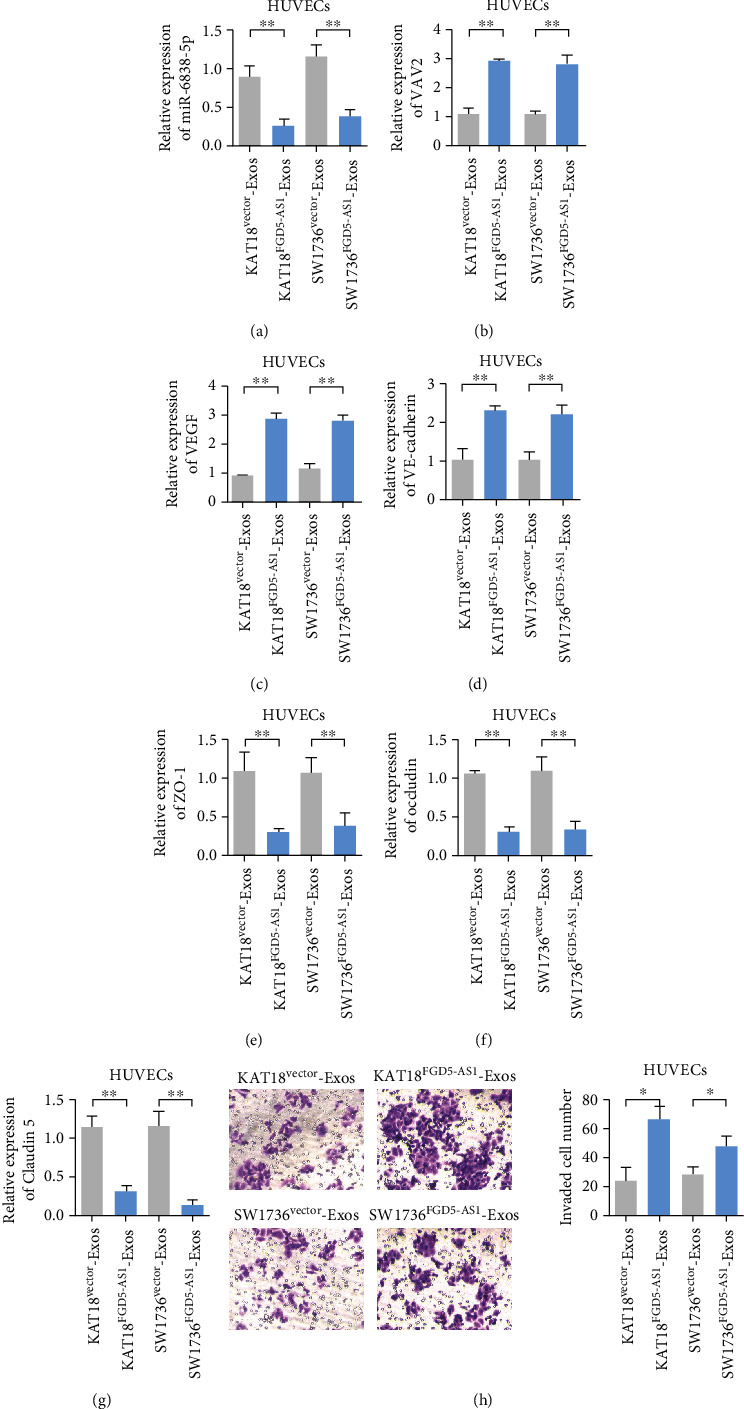
Exosomal FGD5-AS1 from TC SW1736 and KAT18 cells promotes angiogenesis in vitro. HUVECs were cocultured with SW1736/FGD5-AS1 exosomes, SW1736/NC exosomes, KAT18/FGD5-AS1 exosomes, and KAT18/NC exosomes. (a) Detection of miR-6838-5p expression in HUVECs after different treatments. (b) Detection of VAV2 expression after different treatments. (c) Detection of VEGF expression after different treatments. (d) Detection of VE-cadherin expression after different treatments. (e) Detection of ZO-1 expression after different treatments. (f) Detection of occludin expression after different treatments. (g) Detection of claudin5 expression after different treatments. (h) Effects of coculture of HUVECs with SW1736/FGD5-AS1 exosomes, SW1736/NC exosomes, KAT18/FGD5-AS1 exosomes, and KAT18/NC exosomes on migration. Data are presented as mean ± SD (^∗^*P* < 0.05; ^∗∗^*P* < 0.01).

HUVECs were cocultured with SW1736/FGD5-AS1 exosomes, SW1736/NC exosomes, KAT18/FGD5-AS1 exosomes, and KAT18/NC exosomes. The results showed that HUVEC cells were incubated with the exosome extracted from the transfected FGD5-AS1 overexpressed plasmid and miR-6838-5p expression was decreased, while VAV2 expression was upregulated (Figures 6(a) and 6(b)). The expression levels of angiogenesis markers VEGF and VE-cadherin in SW1736 and KAT18 cells incubated with the exosome overexpressing FGD5-AS1 were upregulated compared with normal control HUVEC cells (Figures 5(c) and 5(d)), while the expression levels of vascular permeability markers ZO-1, occludin, and claudin5 were downregulated (Figures 5(e)–5(g)). Effects of coculture of HUVECs with SW1736/FGD5-AS1 exosomes, SW1736/NC exosomes, KAT18/FGD5-AS1 exosomes, and KAT18/NC exosomes on migration were observed. Results showed that exosome FGD5-AS1 from TC SW1736 and KAT18 cells promoted the migration ability of HUVEC cells (Figure 6(h)).

### 3.7. Exosome FGD5-AS1 Promotes Angiogenesis and Proliferation In Vivo

**Figure 7:**
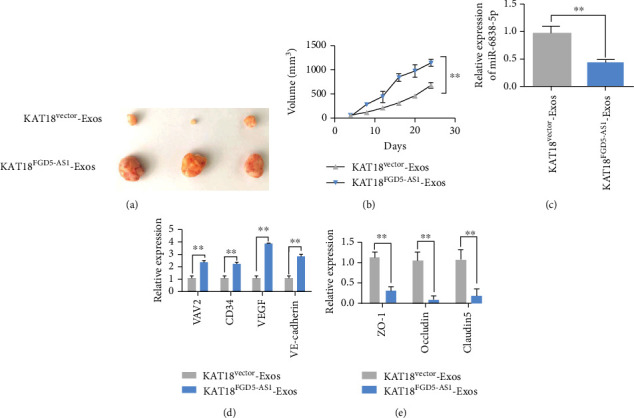
Exosomal FGD5-AS1 promotes angiogenesis and proliferation in vivo. (a) Seven days after subcutaneous tumor bearing, mice xenografts were injected with KAT18^vector^-Exos or KAT18^FGD5-AS1^-Exos (*n* = 6), respectively. (b) Tumor volume after different treatments. (c) The expression level of miR-6838-5p in nude mice was detected by qRT-PCR. (d) The expression levels of VAV2, CD34, VEGF, and VE-cadherin in nude mice were detected by qRT-PCR. (e) The expression levels of ZO-1, occludin, and claudin5 in nude mice were detected by qRT-PCR. Data are presented as mean ± SD (^∗∗^*P* < 0.01).

To construct a subcutaneous model of thyroid carcinoma in nude mice, after 7 days of subcutaneous tumor bearing, subcutaneous xenograft mice were injected with KAT18 ^Vector^-Exos or KAT18^FGD5-AS1^-Exos, respectively. The experimental results showed that the tumor volume in the KAT18^FGD5-AS1^-Exos group was larger and the proliferation rate was faster (Figures 7(a) and 7(b)). The expression level of miR-6838-5p in nude mice was detected by qRT-PCR. The expression level of miR-6838-5p in tumor tissues of the KAT18^FGD5-AS1^-Exos group was downregulated (Figure 7(c)). In addition, we found that the expression levels of VAV2, CD34, VEGF, and VE-cadherin in tumor tissues of the AKT18^FGD5-AS1^-Exos group were upregulated (Figure 7(d)). However, the expressions of ZO-1, occludin, and claudin5 in tumor tissue specimens of nude mice injected with KAT18^FGD5-AS1^-Exos were downregulated (Figure 7(e)).

## 4. Discussion

TC is one of the most common endocrine tumors [20]. The biological behavior and prognosis of TC vary greatly among different histological types. At present, the efficacy of traditional treatment for highly aggressive TC is still very limited [21]. Tumor angiogenesis provides oxygen and nutrients for tumor tissue metabolism [22]. At the same time, it can provide energy for distant metastasis of tumor and promote tumor growth and metastasis [23]. Tumor angiogenesis involves a variety of cells and molecules and is affected by many factors. Vascular endothelial growth factor (VEGF), basic fibroblast growth factor (BFGF), matrix metalloproteinase (MMP), epidermal growth factor receptor (EGFR), and angiopoietin are important factors promoting tumor angiogenesis and play an important role in the development and progression of tumor [24, 25].

Exosomes can transfer their transport molecules into the cytoplasm of target cells through endocytosis [26]. Through the interaction between receptors and target cells, the downstream intracellular signaling pathway is activated. Exosomes can be produced by endosomes of various cells, including tumor cells [27]. Exosomes can induce angiogenesis and transmit gene information by modulating immune response [28]. Exosomes integrate information during tumor cell proliferation and metastasis to promote tumor growth and metastasis. The inclusions of exosomes include protein, mRNA, miRNA, lncRNA, and other macromolecules. Under different stress states, the number and inclusions of exosomes produced by cells are correspondingly different [29]. According to the central rule, the transfer of nucleic acids in exosomes may have a greater biological effect than the transfer of proteins. lncRNA can regulate recipient cells, promote tumor metastasis, drug resistance, cellular metabolic reprogramming, increased tumor stemness, epithelial-mesenchymal transformation (EMT), angiogenesis, and lymphangiogenesis, and induce immunosuppression [30]. In-depth understanding of the role of tumor exosomal lncRNA in tumor microenvironment is helpful to provide new diagnostic markers and clinical therapeutic targets for tumors.

In this study, secreted exosomes were detected from two in vitro cultured TC cell lines SW1736 and KAT18. Exosomes were cocultured with HUVEC cells, and the angiogenesis ability and vascular permeability were detected. The results showed that exosomes isolated from TC cell lines and cultured with normal HUVEC cell lines increased angiogenesis and vascular permeability of HUVECs. Mechanism studies have shown that the tumor-derived exosome can transfer FGD5-AS1 and then act on the effector miR-6838-5p/VAV2. The preliminary study of FGD5-AS1 transfected with a plasmid showed that the proliferation and migration of HUVEC cells were enhanced after FGD5-AS1 was overexpressed. Therefore, we found that tumor-derived exosome metastatic lncRNAs, including FGD5-AS1, induce tumor angiogenesis and establish a local tumor microenvironment and distant metastasis niche. Therapies that target exosomes secreted by tumor cells and the lncRNAs they carry may reduce the ability of tumors to metastasize.

MicroRNAs (miRNAs) inhibit target gene expression at the posttranscriptional level by complementing the 3′ untranslated region (3′-UTR) of target gene mRNA [31]. miRNA regulates cell differentiation, proliferation, apoptosis, metastasis, and other physiological processes, participates in the occurrence and development of diseases, and is closely related to the formation of tumors [32]. Liu et al. [33] found that the expression level of miR-6838-5p was reduced in triple negative breast cancer. Regulation of downstream target gene expression affects the activation of the Wnt signaling pathway and inhibits the proliferation and epithelial mesenchymal transformation of triple negative breast cancer cells. Low expression of miR-6838-5p promotes the proliferation and metastasis of triple negative breast cancer cells. miR-6838-5p showed tumor suppressive activity. This study found that the relative expression level of miR-6838-5p in TC tissues was lower than that in paracancer tissues. In addition, miR-6838-5p was found to be the target gene of FGD5-AS1. The prediction of TargetScan software indicated that VAV2 might be the target gene of miR-6838-5p. The targeting relationship between miR-6838-5p and VAV2 was further explored. The results of the double luciferase reporter gene assay showed that miR-6838-5p and VAV2 mRNA had complementary binding sites. miR-6838-5p can bind with VAV2 mRNA in a targeted way.

Tumor angiogenesis is associated with tumor growth, invasion, metastasis, recurrence, and prognosis [6, 34]. Tumor microenvironment promotes tumor angiogenesis through various mechanisms under the stimulation of inflammation and inflammatory mediators [5, 35]. The interaction between the tumor and vascular endothelium plays an important role in supporting hematogenous metastasis of tumor [36]. VAV2 is highly expressed in a variety of tumor cells and tissues, including lung cancer, thyroid papillary carcinoma, and breast cancer. VAV2 is involved in the formation of filamentous pseudopodia, lamellar pseudopodia, and membrane folds of tumor cells and regulates the migration and infection of tumor cells [37–39]. Activation of the phosphorylated form of VAV2 has been reported in highly invasive tumor cell lines. Transfection of activated VAV2 into the immortalized keratinocyte cell line HaCat and invasive cell line OSCC expressing VAV2 at a low level can enhance the invasion ability of cells [40]. RhoC is a member of the Rho family of genes, which are key genes that regulate the cytoskeleton (actin). It is involved in many important life activities such as cell differentiation, proliferation, and migration. Among many upstream regulatory factors of Rho, the VAV family plays a particularly important role. VAV regulates actin cytoskeleton by activating the Rho/Rac signaling pathway. Moreover, this effect is central to the network of actin-related signaling pathways. It has been confirmed that VAV proteins are involved in many life processes that require cytoskeleton reconstruction. This study found that TC exosomes regulate the miR-6838-5p/VAV2 axis through FGD5-AS1, promoting tumor angiogenesis and vascular permeability.

## 5. Conclusion

In this study, we confirmed that exosomes derived from TC mediate the important role of FGD5-AS1 in the development and progression of TC. We found that exosome FGD5-AS1 promotes angiogenesis, vascular permeability, and metastasis by targeting the miR-6838-5p/VAV2 axis in vascular endothelial cells. The overexpression of FGD5-AS1 in exosomes isolated from peripheral blood of patients with TC may contribute to the prognosis of TC and provide a new reference for clinical diagnosis and treatment of TC.

## Data Availability

The data used to support the findings of this study are available from the corresponding author upon request.
